# Ancient schwannoma involving the median nerve: a case report and review of the literature

**DOI:** 10.1007/s11751-013-0158-7

**Published:** 2013-04-02

**Authors:** Konstantinos Malizos, Maria Ioannou, Vasileios Kontogeorgakos

**Affiliations:** 1 Department of Orthopaedic Surgery and Musculoskeletal Trauma, Faculty of Medicine, School of Health Sciences, University Hospital of Thessalia, 41110 Larissa, Greece; 2 Department of Pathology, Faculty of Medicine, School of Health Sciences, University of Thessaly, 41110 Biopolis, Larissa, Greece

**Keywords:** Benign neural sheath tumors, Ancient schwannoma, Neurilimoma, Median nerve

## Abstract

Ancient schwannomas are benign long standing schwannomas of the neural sheaths. Histological findings are these seen as in conventional schwannomas, but ancient schwannomas additionally demonstrate cystic hemorrhagic changes and degenerative nuclei with pleomorphism and hyperchromasia. Due to the nuclear atypia, and cystic degeneration, ancient schwannomas might be confused with malignant tumors on histology and imaging, leading to a radical surgical approach. The median nerve is rarely affected. We present a rare case of an ancient schwannoma involving the median nerve at the mid humerus. The tumor slowly grew up within ten years and become symptomatic with local pain, mild numbness in the distribution of the median nerve in the palm and Tinel’s test. The tumor was successfully removed by separating it from the nerve fascicles to negative margins. Post-operatively local symptoms relieved but minor sensory loss in the median nerve distribution in the palm was noticed which improved in the following six months. Ancient schwannomas can be misdiagnosed as sarcomas due to specific imaging and histologic findings. Patients’ physical examination, history and fine radiologic and pathology features should be cautiously interpreted in order to achieve correct diagnosis and avoid unnecessary wide tumor excisions.

## Introduction

Schwanomas, also known as neurilemomas, are the most common benign tumors of the neural sheaths of the peripheral nerves [1]. Although they commonly appear as solitary lesions, occasionally there can be multiple (shcwannomatosis) or an association with neurofibromatosis [2, 3]. Very rarely they undergo malignant transformation [2, 4]. Usually the ulnar and peroneal nerve are involved while only 7 % of schawnnomas involve the median nerve [5]. Ancient schwannomas were originally described in 1951 by Ackerman and Taylor [6]. They are rare long standing tumors that have undergone degenerative changes, accounting for 0.8 % of soft tissue tumors. Their histology findings are those seen in schwannomas, with the additional features of degeneration and mild nuclear atypia that can pose diagnostic dilemmas from the malignant counterpart [1, 6].

Here we report on a very rare case diagnosed as ancient schwannoma of the median nerve. We describe the clinical presentation and the specific imaging, histology, surgical findings and functional outcome. We discuss the rationale of the approach of such a tumor, posing diagnostic and treatment difficulties.

## Case report

A 40 year old man presented with a painful mass at the medial side of his arm. On clinical examination there was a painless solid mass 6–7 cm in length, little sensitive to pressure, firmly attached to deeper tissues. Percussion over the mass produced a Tinel’s like sensation along the median nerve. The patient experienced mild numbness in the distribution of the median nerve in the palm but no motor weakness or muscle atrophy was detected. No cutaneous pigmented lesions were found. The patient reported he first palpated a nodule 10 years ago that grew up very slowly to the current size. No history of significant trauma was recalled.

**Fig. 1:**
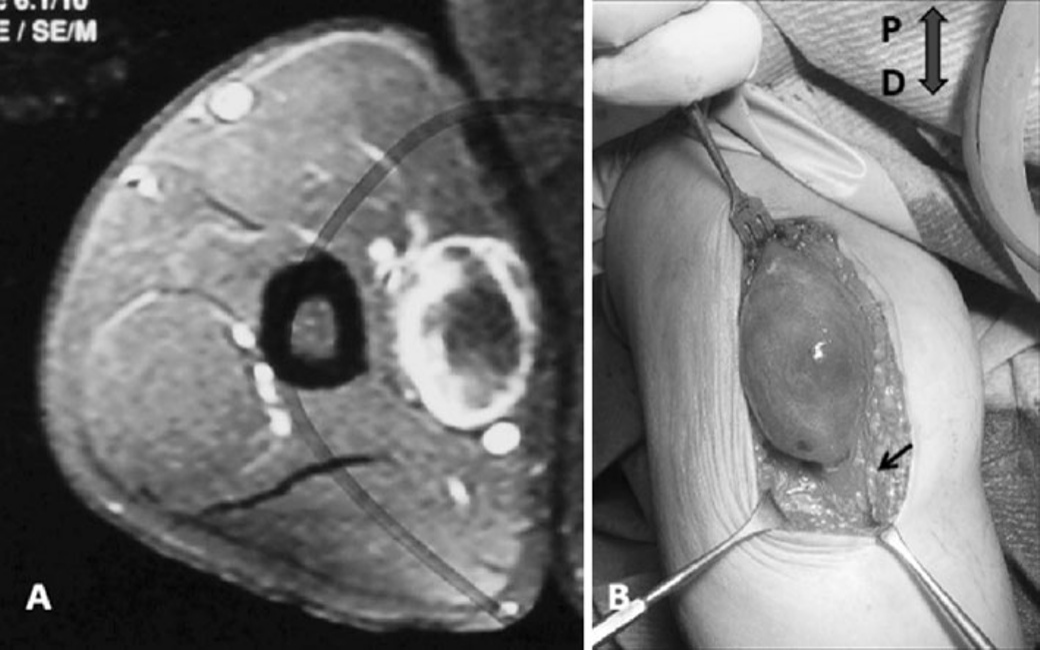
**a** Axial view, fat suppressed with gadolinium. Round lesion well circumscribed with enhancement of the periphery. **b***Oval shaped* capsulated mass in eccentric position along the median nerve (*arrow*). *P* proximal; *D* distal

An MRI examination revealed a 6 cm long, well circumscribed mass in close proximity to the neurovascular bundle of the arm. The lesion had a relatively homogenous low signal, slightly lower to the muscle, on T1 images. On T1 fat suppressed images, increased gadolinium enhancement was noticed at the periphery of the mass with a nonenhancing low signal central area (Fig. 1a). No peri-tumoral edema was present and a line of fat surrounded the tumor. Based on the long history of the patient, and the clinical and MRI features, the diagnosis of a nerve sheath tumor arising from the median nerve was supported and the patient was scheduled for excisional biopsy. A longitudinal incision centered over the tumor bulk was performed. The tumor had an eccentric position and was firmly attached to the median nerve, (Fig. 1b). An incisional biopsy was performed and tissue sample was taken from the proximal pole of the mass (where more viable tissue was seen on MRI). The sample was considered to be representative of the entire pathology macroscopically and was sent for frozen section. Intra-operative biopsy examination revealed nuclear atypia. Howeve, significant mitotic activity was not seen and the lesion was characterized as a benign peripheral nerve sheath tumor. The decision to preserve the median nerve and perform a marginal tumor excision was undertaken. Under high power magnification, the epineurium was longitudinally incised and the soft tumor mass dissected from surrounding nerve fascicles. Internal neurolysis started from the poles of the mass and a few nerve fascicles entering to the mass had to be sacrificed. Immediately post-operatively the patient experienced minor sensory loss in the median nerve territory of the palm without noticeable weakness of the muscles innervated by the median nerve. At 6 months follow up the wound is well healed with no pain upon palpation. The upper extremity is neurologically intact except for the partially improved numbness mainly in the long finger. The patient returned to full labor work as a fisherman.

**Fig. 2:**
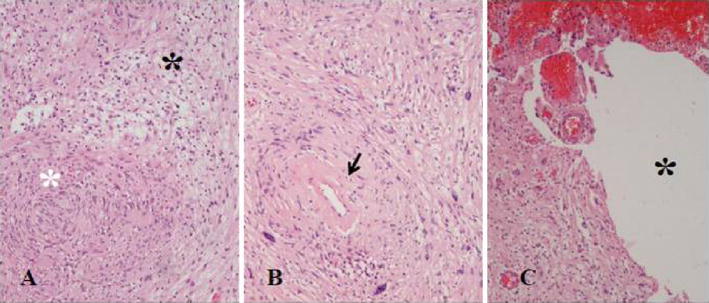
Hematoxylin and Eosin stain, original magnification ×10, **a** Alternating Antoni A (*white asterisk*) and Antoni B (*black asterisk*) patterns, **b** Hyalinized and thickened blood vessel (*arrow*). **c** Degenerative changes. *Black asterisk*: cystic area

The final gross pathology evaluation demonstrated an ovoid tumor measuring 5.5 × 3 × 2.5 cm. The center of the mass was cystic filled with a brown colored fluid. Histologically there were alternating hypocellular and hypercellular areas (Fig. 2). The hypercellular areas showed elongated spindle cells with nuclear palisading forming occasional Verocay bodies, whereas the hypocellular areas showed loosely arranged neoplastic cells in a myxoid background with hyalinized thick-walled blood vessels and foci of hemorrhage with hemosiderin-laden macrophages. In these areas significant nuclear atypia and pleomorphism with multinucleation and nuclear invaginations were seen, however mitoses were scarcely found and atypical mitotic figures or necrosis was not observed. The S-100 stain was positive. Immuno-staining with the proliferation index Ki67 (MIB-1) displayed a low proliferating index. The final pathology report was consistent with ancient schwannoma.

## Discussion

Schwannomas can be asyptomatic or can produce pain, a positive Tinel’s sign or a Tinel’s like sensation and sensory alterations [5, 7]. The slow growth pattern of benign nerve tumors, allows for adaptation of the nerve function to the pressure effects. Thus, although schwannomas can rarely induce impaired motor function, neural tumors producing motor deficits should always raise a high suspicion of malignancy [7].

On pathologic analysis, a schwannoma has a true capsule composed of epineurium. The hallmark of schwannomas is the alternating pattern of Antoni A and B areas [1]. Antoni A are cellular areas with spindle cells and nuclear palisading forming Verocay bodies whereas Antoni B are hypocellular areas in a myxoid background. Ancient schwannomas display pronounced degenerative changes attributed to the vascular insufficiency during growth of the tumor and the tumor size has been correlated with progressive degenerative features [8]. They are characterized by cystic necrosis, stromal edema, xanthomatous change, fibrosis, perivascular hyalinization, calcification and degenerative nuclei with pleomorphism and hyperchromasia [1, 6, 9]. Antoni A and Antoni B areas are also observed as in conventional schwannomas. There is, however, a relative loss of cellular regions, which tend to be fibrosed or sclerotic. These areas may degenerate into hematomas and cysts [1, 9]. Nuclear atypia, and hyperchromasia, however, with low mitotic rate, can mislead to the diagnosis of malignancy.

On MRI the morphology and signal intensities reflect the histologic components of schwannomas. A rim of low intensity surrounds the lesion corresponding to the epineural capsule [10]. Antoni A areas are cellular areas containing collagen, thus exhibiting low signal in T1 and T2 images. In Antoni B, the water containing myxoid matrix produces a low in T1 and high signal intensity in T2 images [10]. In conventional schwannomas, central location of the Antoni A areas produces the so-called target sign on T2 images [7]. Cystic and necrotic areas have low signal in T1 sequences and are not enhanced with gadolinium [10]. These heterogeneous signal areas combined with cystic formations are easily interpreted as aggressive features seen in malignant peripheral nerve sheath tumors or other high grade sarcomas.

In our case, the cystic areas involved a higher percentage compared to the viable tumor on an axial view. On MRI the viable tumor areas were enhanced after gadolinium administration and, because of their peripheral location, a bright ring surrounded the nonenhancing irregular central cystic areas.

As a rule, biopsy of a soft tissue mass should be performed before surgical treatment is scheduled. Closed biopsy techniques are highly diagnostic with a low complication rate [11]. However, biopsy of a nerve tumor can cause neuropathic pain [5]. Excision biopsies should be performed only when imaging studies reveals a mass that can be completely excised and the surgeon considers that it is going to be the final surgical procedure. It is well known that unplanned excision biopsies are associated with a high rate of residual tumor in the surgical bed [12]. In this case, clinical examination in association with MRI features was suggestive of a long standing peripheral nerve tumor with possible diagnosis of a malignant peripheral nerve sheath tumor vs degenerated schwannoma. In our case, a closed biopsy could be either nondiagnostic or false positive for malignancy due to the extensive cystic areas and atypical histological features commonly seen in degenerated, old neural sheath tumors. Based on the fact that the lesion was well defined on MRI and frozen sections were negative for malignancy, we decided to perform a marginal tumor resection to the negative histological margins. So, even in the worst scenario, if the final pathology diagnosed a malignant tumor, the negative intra-operative margins protected the patient from contamination of the surgical bed from tumor remnants.

In contrast to neurofibromas and primary malignant nerve sheath tumors, schwannomas are eccentrically placed in relation to the axis of a large peripheral nerve, well encapsulated and can be dissected from the fascicles of the parent nerve without jeopardizing permanent or severe nerve dysfunction [5, 13]. Minor nerve dysfunction after schwannoma excision can be the result of three possible reasons [14]. The first is neuropraxia from nerve handling during tumor removal. In these cases nerve function recovers within weeks. The second reason is that small nerve fibers running through the capsule of the tumor are divided during capsule opening. In these cases a minor nerve function loss is noted. The third reason is that schwannomas arises from neural sheaths and the fascicles surrounded by this part of the sheath may have to be rejected. The resultant deficit depends on the specific function of this fascicle. Additionally, there are few cases were schwannomas demonstrate an infiltrative pattern and tumor excision necessitates some fascicle resection. Sawada et al. [14] demonstrated that schwannomas that could not be enucleated were those located proximal to the middle upper extremity and pre-operatively had sensory disturbances. When motor function loss is expected by intra-operative nerve fascicle stimulation, nerve grafting is recommended [15]. Tumor recurrence is rare, even in incomplete resections [15].

Here we present a rare example of a longstanding, symptomatic ancient schwannoma of the median nerve. Histology findings from small tissue sampling and MRI features may suggest malignancy leading to unnecessary wide tumor excision with loss of nerve function. Careful evaluation of the patient’s history with the relatively long time for existence of the nodule and the subtle clinical findings, in association with the specific frozen section histology findings and imaging features, are essential for intra-operative decision making to avoid unnecessary sacrifice of the parent nerve.
